# The complete mitogenome of Qingyuan chicken (Gallus gallus) by next-generation sequencing and phylogenetic analysis

**DOI:** 10.1080/23802359.2020.1791757

**Published:** 2020-07-20

**Authors:** Jingjing Gu, Sheng Li

**Affiliations:** a College of Animal Science and Technology, Hunan Agricultural University, Changsha, China; b Hunan Key Laboratory for Genetic Improvement of Animals, Changsha, China; c Hunan Engineering Research Center of Poultry Production Safety, Changsha, China; d Maxun Biotechnology Institute, Changsha, China

**Keywords:** Qingyuan chicken, phylogenetic analysis, next-generation sequencing

## Abstract

In this study, the complete mitochondrial genome sequence of the Qingyuan chicken (Gallus gallus) was reported for the first time by using the high-throughput sequencing technology. The complete mitogenome sequence of Qingyuan chicken exhibits a typical genetic structure of vertebrate double-strands circular genome, containing a D-loop region, 2 ribosomal RNA genes, 13 protein-coding genes and 22 transfer RNA genes. Our work provides a source of useful data for mitochondrial researches and chicken phylogenetic studies.

Qingyuan chicken is one of the famous indigenous chicken breeds in China and granted as a National Geographical Indication Product. This chicken is renowned for its excellent meat quality and plays an important role in Guangdong cuisine. The appearance of Qingyuan chicken can be described as a wedge-shaped body with compact front body part and roundness rear one. Qingyuan chicken also has a thin head and slim feet with parti-colored feathers. In this study, we sequenced and assembled the complete mitochondrial genome of Qingyuan chicken using next-generation sequencing technology for the first time. Qingyuan chicken sample was obtained from Qingyuan City (23.70 N and 113.03 E), Guangdong province, China. The Qingyuan chicken specimen (Voucher No. QY150882) was stored at −80 °C in the Museum of Hunan provincial key laboratory for genetic improvement of domestic animal, Changsha, China for long-term usage. The raw sequencing reads of Qingyuan chicken were 11.22 Gb in total and were uploaded onto NCBI Sequence Read Archive (SRA) with accession number SRR4302057. The assembled complete mitochondrial genome of Qingyuan chicken has been deposited in Genbank with accession number MT635913.

The complete mitochondrial genome sequence of Qingyuan chicken has been annotated by tRNAscan-SE 2.0 (Chan and Lowe 2019) and MITOS (Bernt et al. 2013) using the full length of 16,784 bp. This mitogenome has a typical double strand circular structure, containing one D-loop region, 2 ribosomal RNA genes (rRNAs), 13 protein-coding genes (PCGs), and 22 transfer RNA genes (tRNAs). Most genes including 2 rRNAs, 12 PCGs and 14 tRNAs are encoded on the heavy chain, while the rest genes (1 PCG and 8 tRNAs) are encoded on the light chain. Twelve out of thirteen PCGs are initiate with an ATG start codon except for *COX1*, which begins with GTG. There are four types of stop codons which are TAA, TAG, AGG and incomplete stop codon T, which is due to the 5′ terminal of adjacent gene (Anderson et al. 1981).

The complete mitogenome of the Qingyuan chicken together with other downloaded chicken mitogenomes are used to generate the neighbor-joining (NJ) phylogenetic tree using Mega 7.0 (Kumar et al. 2016) with 1000 bootstrap replicates. The NJ tree (Figure 1) shows the Qingyuan chicken has the closest maternal relationship with Xuefeng, Huaiyang and Nandan. Also, Qingyuan chicken is close related with Xianju, Tibetan and Daweishan Mini. However, Lverwu has the furthest genetic distance with Qingyuan chicken. Our work provides a source of useful data for mitochondrial researches and chicken phylogenetic studies.

**Figure 1.**
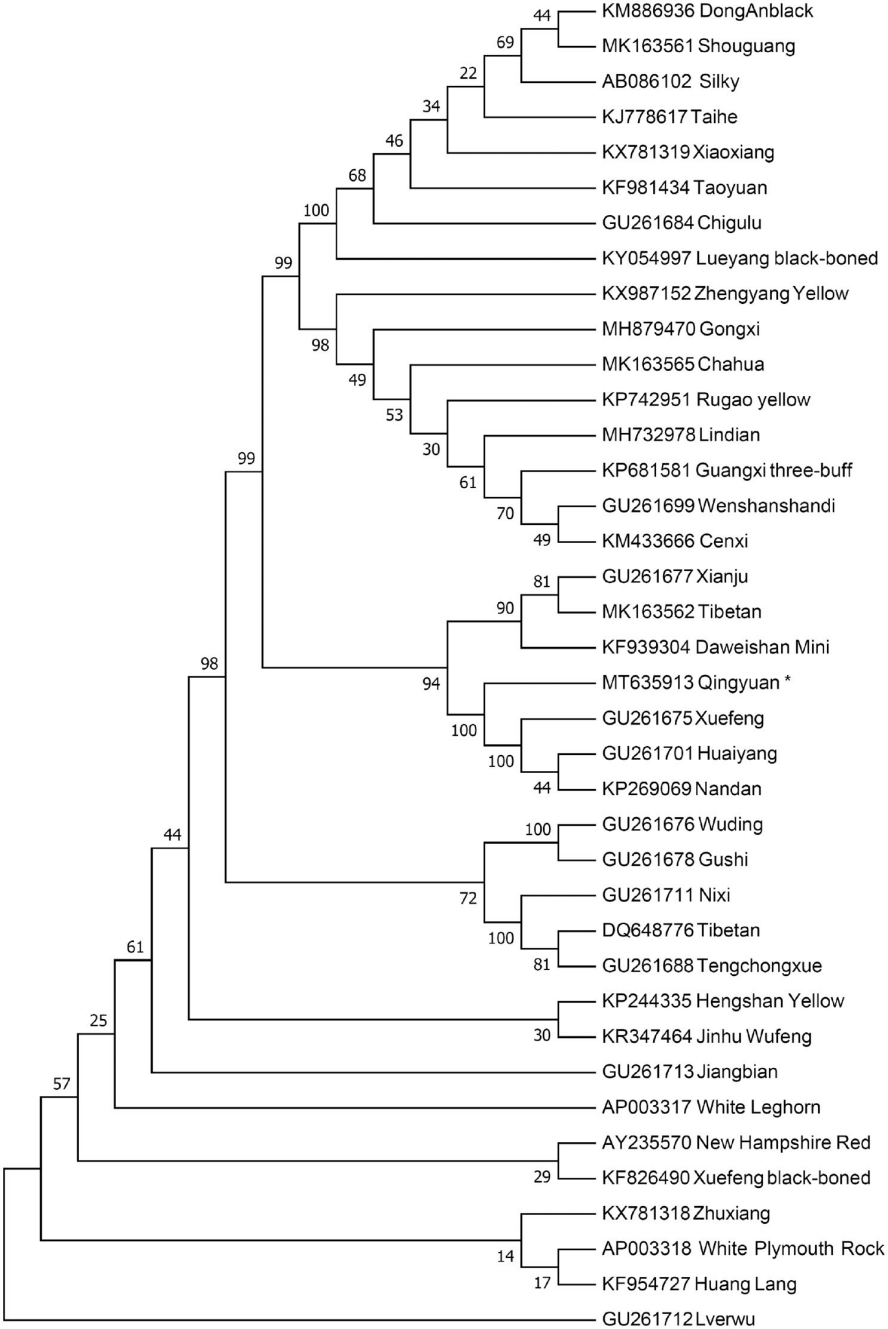
Neighbor-joining tree based on the complete mitochondrial DNA sequence of 38 chicken breeds. GenBank accession numbers are given before the species name.

## Data Availability

The sequence data that support the findings of this study are openly available in the NCBI Sequence Read Archive (SRA) at http://www.ncbi.nlm.nih.gov/sra/ with accession number SRR4302057. The complete mitochondrial genome of Qingyuan chicken (Gallus gallus) is openly available in GenBank at http://www.ncbi.nlm.nih.gov/genbank with accession number MT635913.
